# A Psychiatrist with Postoperative Anxiety After Hysterectomy: How Could This Be Fatal?

**DOI:** 10.7759/cureus.3909

**Published:** 2019-01-17

**Authors:** Larry Nichols, Ashley Reavis McNeal, Keisha Callins

**Affiliations:** 1 Pathology, Mercer University School of Medicine, Macon, USA; 2 Obstetrics and Gynecology, Mercer University School of Medicine, Macon, USA

**Keywords:** patient safety, ileus, asthma, physician as patient, autopsy, history and physical examination, postoperative care, abdominal surgery

## Abstract

Hysterectomy is a common surgery and rarely fatal. Here, we present a case of hysterectomy with postoperative complications, leading to a fatal outcome. The autopsy revealed the cause of death and clinicopathologic correlation suggested multiple lessons for patient safety. Patient safety can be enhanced by appropriate boundaries in the care of physician-patients, proactive monitoring and prompt treatment of postoperative asthma, nasogastric suction for postoperative ileus associated with vomiting, and assessment of the patient situation by a good history and physical examination, going to see the patient in person.

## Introduction

Hysterectomy is a common surgery and rarely fatal [1]. When the outcome of a routine hysterectomy is a fatality, a thorough investigation, including an autopsy, with careful clinical and pathologic correlation, is warranted to formulate a root cause analysis and search for ways to improve the quality of care and post-surgical outcomes for future patients. This case is reported for these purposes.

## Case presentation

A 49-year-old woman was admitted for an elective abdominal hysterectomy. She was an unmarried psychiatrist originally from Argentina, living in Northeast USA. She had an 11-year history of asthma and a history of hypertension. Her surgical history included tonsillectomy at age 11, right carpal tunnel release at age 37, cholecystectomy for cholelithiasis at age 41, right breast nodule excised at age 45, which showed intraductal hyperplasia, adenosis and chronic cystic mastitis (fibrocystic change), and cervical laminectomy at age 47. Her social history included a 10 pack-year history of smoking ending at age 32 and no history of alcohol use.

On admission, she was afebrile, with a pulse of 96 beats/minute, blood pressure of 168/104 mmHg, respirations of 20 breaths/minute, and obesity (body mass index 35.5 kg/m2). Her chest was clear and she had a 14 cm "nodular fibroid uterus." Preoperative white blood cell count was 10,300/cu mm, hematocrit 36.8%, potassium 3.7 mEq/L, sodium 142 mEq/L, blood urea nitrogen 11 mg/dL, and urinalysis negative. Chest X-ray showed "poor inspiratory effort with low lung volumes." The electrocardiogram showed small Q waves in the inferior leads.

The patient underwent a total abdominal hysterectomy with bilateral salpingo-oophorectomy. The surgical specimen showed a 5 cm leiomyoma, endometriosis, and a hemorrhagic corpus luteum of the right ovary. The first postoperative day was uneventful. On the second postoperative day, the patient developed postprandial nausea and vomiting relieved with prochlorperazine. The abdominal X-ray showed an ileus. The patient's potassium was 3 mEq/L. This was corrected to 4 mEq/L but the ileus persisted.

On the third postoperative day, repeat chest X-ray showed atelectasis despite incentive spirometry. That night, the patient had insomnia, which she had also suffered each of the previous three nights. There was a night shift nurse on duty each of these nights, who helped the patient pass the time, talking to her when she was not busy with other patients. That night, at midnight, the nurse took the patient's vital signs: her temperature was 39.1 degrees C (102.4 degrees F), pulse 88 beats/minute, blood pressure 160/92 mmHg, and respiration 24 breaths/minute.

At 01:00, the patient told the nurse that she had slight surgical incision pain and emesis. The nurse paged the obstetrics/gynecology resident on call, who gave an order over the phone for oxycodone/acetaminophen and calcium carbonate. This was given.

At 02:00, the patient told the nurse that she was having an anxiety reaction. In fact, the patient told the nurse "I'm really nervous because I am going to die tonight." The patient told the nurse she needed a benzodiazepine and suggested one, with a specific dose. The nurse again paged the resident on call and told him that the patient was having an anxiety reaction and wanted a benzodiazepine sedative. The resident gave an order over the phone for diazepam. This was given.

At 03:40, the patient told the nurse that she was experiencing progressive dyspnea. Her temperature was 38.0 degrees C (100.4 degrees F), pulse 118 beats/minute, blood pressure 118/70 mmHg, and respirations 24 breaths/minute. The nurse again paged the resident on call, who gave an order over the phone to get a respiratory therapist to administer inhaled bronchodilator therapy. There were very few respiratory therapists on duty and the soonest one of them could get to this patient was 40 minutes later.

At 04:20, when the respiratory therapist arrived, the patient had respiratory distress with wheezing and a respiratory rate of 36/minute. She was diaphoretic, cold, and clammy. Her blood pressure was 100/60 mmHg. At 04:30, inhaled bronchodilator therapy was only partially successful in relieving the patient. The nurse again paged the resident on call who gave an order over the phone for intravenous aminophylline therapy.

At 05:00, an attempt by the nurse to start an intravenous line for aminophylline therapy was unsuccessful. While a second attempt was underway, the patient began vomiting large amounts of bilious yellow-green fluid. Shortly after this, the patient suffered a respiratory arrest. Cardiopulmonary resuscitation was begun, but it was to no avail and the patient was pronounced dead at 06:15.

The postmortem examination revealed small amounts of freshly aspirated gastrointestinal contents within the tracheobronchial tree and within lower lobe alveoli. In addition, the autopsy demonstrated mucoid fluid secretions in the bronchi, bilateral areas of atelectasis, patchy acute bronchitis, and pneumonia within the lower lobes, more on the right side. The stomach and entire length of small and large intestines were massively dilated. The liver had diffuse marked steatosis.

## Discussion

Together, the clinical history and autopsy findings suggest that the chain of events leading to this patient’s death included postoperative ileus, which led to vomiting, aspiration, and subsequent pneumonia, which combined with an asthma attack, caused by the irritative effect of aspiration into the bronchi. Knowing the chain of events that eventuated in this patient’s death, one can draw multiple lessons for patient safety from this case. Perhaps, foremost is the importance of assessing a patient situation in person, going to see the patient. At 01:00, when the patient had vomiting and the nurse called the resident, the patient had fever and tachypnea an hour earlier, presumably manifestations of the pneumonia she had six hours later, demonstrated by the autopsy. It is possible that if the resident had gone to see the patient at 01:00, he might have diagnosed pneumonia and started antibiotic therapy, potentially changing the outcome. At 03:40, when the patient had progressive dyspnea and the resident ordered treatment for asthma over the phone, if he had gone to see the patient, he might have appreciated the severity of her illness and started more aggressive therapy, again potentially changing the outcome. The analysis of this case and others [2] suggests that likely nothing is better for patient safety than good in-person history and physical examination.

The first in the chain of events leading to this patient’s death was postoperative ileus, which led to vomiting and persisted despite the correction of hypokalemia. The failure of peristalsis leads to the accumulation of secretions in the gastrointestinal lumen and removing them can decrease the risk of these secretions overflowing into the respiratory tract. It is possible that nasogastric suction might have prevented enough of the vomiting and aspiration to have changed the outcome in this case. The analysis of this case suggests that nasogastric suction to reduce the risk of vomiting and aspiration in patients with ileus may sometimes be a valuable measure to improve patient safety [3].

An asthma attack played a prominent role in the fatal outcome in this case. Surgery in patients with asthma carries a risk of serious adverse outcomes. In a study of 24,109 surgical patients with asthma, they were 48% more likely to have postoperative pneumonia, 11% more likely to develop septicemia, and 9% more likely to die [4]. The patient, in this case, had a history of asthma. It is worth considering whether an order for regular monitoring by respiratory therapy and a standing order for nebulizer treatments, as needed, might have changed the outcome in this case. Proactive monitoring for an exacerbation of asthma and prompt intervention may sometimes be valuable measures to improve postoperative patient safety [5].

 The psychiatrist-patient, in this case, may have contributed to her own demise by asking for a respiratory depressant sedative in the context of aspiration, pneumonia, and asthma. Caregivers often assume that a physician-patient is better able to recognize truly threatening symptoms and be an informed self-advocate than is actually the case. Specialist physician-patients may pose some of the greatest challenges in their own care. The danger is especially acute when care is being directed by a junior physician or physician in training and the patient is a senior physician. It is important for the safety of physician-patients to establish appropriate boundaries with predefined roles in the medical care management plan [6].

## Conclusions

Routine abdominal surgery can be complicated by postoperative ileus, vomiting, and aspiration, resulting in a life-threatening combination of pneumonia and asthma attack. Respiratory depressant medication, ordered in this case by a resident influenced by a physician-patient, can worsen the threat of asthma or pneumonia. Patient safety can be enhanced by appropriate boundaries in the care of physician-patients, proactive monitoring, prompt treatment of postoperative asthma, nasogastric suction for postoperative ileus associated with vomiting, and going to see the patient in person to assess the situation by a good history and physical examination. The application of these lessons from this case could make the difference between life and death.
